# Unveiling the hidden burden: Exploring the psychosocial impact of cutaneous leishmaniasis lesions and scars in southern Ethiopia

**DOI:** 10.1371/journal.pone.0317576

**Published:** 2025-02-05

**Authors:** Behailu Merdekios, Misgun Shewangizaw, Abebayehu Sappo, Eshetu Ewunetu, Johan van Griensven, Jean-Pierre van geertruyden, Melissa Ceuterick, Hilde Bastiaens

**Affiliations:** 1 Department of Public Health, Arba Minch University, Arba Minch, Ethiopia; 2 Department of Social Anthropology, Arba Minch University, Arba Minch, Ethiopia; 3 Institute of Tropical Medicine, Antwerp, Belgium; 4 Global Health Institute, University of Antwerp, Antwerp, Belgium; 5 Health and Demographic Research, University of Ghent, Ghent, Belgium; 6 Department of Family Medicine and Population Health, University of Antwerp, Antwerp, Belgium; Kerman University of Medical Sciences, ISLAMIC REPUBLIC OF IRAN

## Abstract

**Background:**

Cutaneous leishmaniasis (CL) poses a major public health concern in Ethiopia, with lesions and scars commonly affecting exposed body parts, resulting in physical, social, and psychological consequences. This study aims to assess the psychosocial impacts of CL, shedding light on the experiences and perceptions of affected individuals, thus contributing to the knowledge on Cutaneous leishmaniasis in Ethiopia and informing public health interventions to address its psychosocial effects.

**Methods:**

Using a descriptive phenomenological design, the study explored the lived experiences of individuals with Cutaneous leishmaniasis lesions and scars. Participants were purposively selected, and data was collected through open-ended in-depth interviews. The analysis combined inductive and deductive approaches through an iterative process, developing a coding framework with seven themes (lesion & CL scar each) and subthemes, resulting in giving important insights in the psychosocial impacts of CL. NVivo 12v supported the analysis process.

**Result:**

The study unveiled negative views and misconceptions surrounding CL and its impact. Application of traditional herbal medicine for CL lesions often leads to pus formation and a foul odour, triggering negative attitudes from others, resulting in embarrassment, pain, and anxiety, leading to discomfort and isolation. The negative psychosocial attitudes associated with CL scars deeply impacted affected individuals, influencing their behaviour. This included isolation and absenteeism from school. CL scars served as unique identifiers, shaping the affected individuals’ identity and self-perception. The unreceptive environment affected the participant’s self-esteem and coping mechanisms. The negative impact of CL scars extended to role performance, marriage prospects, and overall happiness, particularly for females facing additional societal pressure and stigma.

**Conclusion:**

The study highlights the need for improved education and awareness about CL to reduce misconceptions and negative attitudes towards affected individuals. Additionally, more effective treatment options and integrated preventive ways should be explored to minimize the physical and psychological impact of CL on affected individuals.

## Introduction

Neglected Tropical Diseases (NTDs) are a diverse group of 20 conditions that are mainly prevalent in tropical areas, where they mostly affect impoverished communities and disproportionately affect women and children. These diseases cause devastating health, social and economic consequences to more than one billion of the global community. Cutaneous Leishmaniasis (CL) is the most common form of leishmaniasis, a group of parasitic diseases caused by the *Leishmania* parasite that has increasingly become a public health concern globally and according to the latest report of the World Health Organization (WHO), 98 countries are endemic for CL and more than a million new cases of CL occur annually [1,2].

Cutaneous leishmaniasis is a skin disease transmitted by the bite of phlebotomine sandflies. CL can be a disfiguring and stigmatizing condition, especially for the lesions that appear over the visible parts such as the facial lesions [3]. CL when healed can leave permanent scars and cause significant impairment or shame. Among the endemic countries, the Americas, the Mediterranean basin, the Middle East, and Central Asia account for around 95 per cent of all global CL cases and yet researchers agree that the actual reported numbers may underestimate the real burden of the disease [4,5]. According to the most recent reports, the top ten nations with the largest number of estimated CL cases are Afghanistan, Algeria, Colombia, Brazil, Iran, Syria, Ethiopia, North Sudan, Costa Rica, and Peru. These countries account for 70 to 75 percent of the anticipated global prevalence of CL. The disease has a substantial influence on afflicted persons’ and communities’ quality of life and socioeconomic standing [6].

CL is a significant public health problem in Ethiopia, causing serious morbidity and having a profound impact on individuals and communities. CL is endemic in Ethiopia, with the country having the highest burden of the disease in Sub-Saharan Africa (SSA) with an estimated annual incidence of 20–50,000 cases [6–8].

The psychosocial impacts of CL in Ethiopia and Africa have not been extensively studied. The disease, as studies show can cause significant social stigma, leading to social exclusion, discrimination, and psychological distress [9]. The long-term consequences of the disease, such as stigmatization and social exclusion, can have a profound impact on the mental health and psychosocial well-being of affected individuals [10].

The term stigma, as used in much of the literatures, has a global inference showing that it takes place in different countries of the world and is not a particular object [11], that stands alone, but is an outcome of combinations of many factors working together [12,13]. Given the broadness of its nature, it gives rise to a great deal of variation in the usage of the term targeting the same social phenomena.

Stigma, as classified by Bernice A, [14] can be either experiential stigma or action-oriented stigma. The former consists of personal experiences and perceptions of stigma, such as perceived stigma, endorsed stigma, anticipated stigma, received stigma, and enacted stigma while the later stigma refers to the actions and behaviors of individuals or social systems that give or receive stigma, such as public stigma, structural stigma, courtesy stigma, provider-based stigma, and self-stigma [14].

However, there is a lack of comprehensive research on the psychosocial impact of CL in Ethiopia where the evidence can be an eye opening to other CL-affected countries in SSA or elsewhere. A study shows that patients with leishmaniasis may have a higher risk of mental illness, psychosocial morbidity, and a reduced quality of life [10]. This bizarre and hostile disease has made over ten decades since it was first recognized in Ethiopia and nearly half a century in Ochollo, a village where this study was carried out. In various countries CL has different names and meanings such as; “Balkh” and “Kandahar sore” in Afghanistan; “Baghdad boil” and “Basra button” in Iraq; and other countries likewise [15]. Here in Ethiopia the synonym includes “Kuncheer” in the north “Chaja” and “Bolbo” in southern Ethiopia, with no attached meaning to each word. It is important to study the psychosocial impacts of CL to gain insights into the depth of the mental pain, daily experiences and specific challenges faced by affected individuals. This can help contribute to the knowledge of the existence of CL-related stigma in Ethiopia and contribute to what type of public health interventions to use and how to address these psychosocial effects. Addressing the psychosocial impacts of CL is essential for promoting the overall well-being and quality of life of affected individuals. The purpose of this study is to investigate the psychosocial impacts of CL among the residents of Ochollo, a village endemic to CL in southern Ethiopia. The findings aim to enhance the well-being of affected individuals in this area and to serve as a foundation for designing targeted and comprehensive information, education, and communication tools for health education in Ethiopia and in elsewhere.

## Methods

### Setting and design

Ochollo is one of the endemic areas in Ethiopia with CL mainly due to *L*. *aethiopica*. CL was first detected in Ochollo in 1973 [16]. This rural village is located approximately 20 km north of Arba Minch at 6°11’N, 37°41’E lying on the western side of the Ethiopian rift valley.

It has an altitude of approximately 2,100 m in the Southern Nations Nationalities and Peoples’ Regional State (SNNPR) of Ethiopia.

There are approximately 5,000 residents in the village and has one primary, one junior secondary school, and one health post. The village has a topography of hills and slopes creating caves and cervices [17]. The overall landscape of the village is rocky with relatively dense vegetation. The study was conducted between May and August 2020 for CL lesion cases and between July and August 2021 for people with CL scars in Ochollo. The village has three traditional healers of which one is very active with number of cases visit his premises.

### Study design

This is qualitative research using a descriptive phenomenological study design used to describe the individual’s life experiences as described by Colazzi in Morrow *et al*., [18].

### Sampling and data collection

We included a purposive sample of participants who had an active lesion and those with scars were selected purposively [19,20] Those with CL lesions were identified by contacting the traditional healers in the village and all scar cases were contacted from the registry of a previously conducted village-wide house-to-house surveillance done by the author. All the participants with lesions and scar who had an active CL lesion and scar were contacted by their address and were involved in this study. The data was captured using an in-depth interview using an interview guide. All participants were interviewed at their own houses to ensure participants were at ease to be interviewed because of sensitive topics. The language used to interview all the participants was “Gamogna”; the language spoken locally. The first data collection was done by a Master’s class student and the second round by the researcher for those with active CL lesions. We looked at the interviews of the lesion and realized that they didn’t provide rich information and we went back to the field with an adapted guide for lesions and scars. We used a theoretical sampling method and data saturation was achieved after ten active lesions and nine CL scar cases were interviewed [21]. All the interviews were audio recorded using a digital sound recorder being each audio saved in a secure separate audio folder on a laptop. The audio data were transcribed into English and an experienced co-authors (MS & EE) did the proofreading of the transcripts compared to the audio records to ensure no translation fallacies have occurred. Field notes were also referred to for individual audio files while transcribing.

### Data analysis

The analysis was guided by the approach described by Collazi [22] and thematic analysis as described by Braun & Clarke [23]. We took an inductive approach, deriving concepts and themes from the transcripts of participants’ experiences without imposing any preconceived ideas. After reading the transcripts several times, a team of two (BM & MS), one member used NVivo 12 software and the other manually did the inductive coding independently identifying the major topics and themes that emerged from the qualitative data. In line to the research question, we finally assumed a single unified and agreed coding framework, with themes and subthemes as mentioned by Braun & Clarke [23]. It was a recursive process that involved comparing the data to make sense of the emerged concepts. Similar ideas were merged, eventually leading to seven major themes for each lesion and scar data (S1 & S2 Tables). During the process of generating the themes and also in the subsequent next reflective step of our analysis, existing literature [24] was used. During this last analysis step, we tried to reflect on how themes relate to each other. Finally, we came up with illustrative diagrams to explain the process how the negative perceptions evolved that we present in this article [24]. Although there was an overlap between the themes and processes for the lesion and scar group, stories are different. For the lesion cases, it is about today more focusing on how to be healed, while for the scars it is seeing the future life and luck. Therefore, we made and kept separate diagrams that we presented in the result part of this paper and we reflected on similarities and differences in the discussion.

I never had any preconception of existence of stigma in the area before I begin my research on the topic. This helped me to do the data collection and analysis with unbiasedness and wholeheartedly which would give a great transferability and generalizability of the findings.

### Data quality for transcription

Standard operating procedures (SOP) were adopted from the works of others and transcription principles were applied for steadiness [25]. We used two people to independently transcribe the audiotaped interview and a concordance comparison was used to assess the agreement between the two transcripts. We have also used the guidelines on how the transcripts should be contained so that it helps us as a qualitative researcher to make sense of and understand the interviewees’ experiences and perceptions, to also help us to remember what is transcribed, what is not transcribed, and how the transcript is structured that significantly influences the analysis process [23] The language accuracy is cross-checked by a third reviewer (EE). A Consolidated criteria for reporting qualitative research (COREQ; S3 Table) checklist was used to ensure the quality [26].

### Reflexivity and rigor

The mother of the principal investigator of this research was born in the very place where this research was undertaken. This might have influenced participant’s collaboration and input. However, the investigator also had the advantage of being a fluent speaker of the local language and familiar with the community’s culture, he has made the preparations mentally and in terms of research data collection tools intuitively and carefully. Whenever he encountered a metaphor that was difficult to understand, the translator by his side helped him grasp its meaning. Although the investigator had known the village for a long time, he had no preconceptions of the existence of stigma in the area before the study began. This helped him to do the data collection and analysis with an unbiased position and wholeheartedly, which increased his interest in the analysis process. As this is a phenomenological study on a sensitive topic, he used a probing question to decrease the interviewer’s influence in interviewing, transcribing and interpreting the findings [27]. As a researcher, he also took steps to support confirmability and paid attention that the interpretations were rooted in the data provided by participants. First, we used the thematic analysis technique which is useful for reducing researchers’ biases and provides a systematic and thorough analysis of the examined topic [28]. Second, the coding process was undertaken by two researchers and regular reflexive meetings were held with experienced qualitative researchers (HB, MC). Finally, the study employed an inductive (next to deductive) approach to analysis, ensuring that participant narratives, rather than the researcher’s prejudices, mostly informed the research findings.

In addition to reflexivity, the study provides detailed accounts of the research setting, cultural context, and participant experiences. We believe this would help readers to understand the broader context and make their judgments about transferability [27,28].

### Ethical considerations

The Institutional Review Board of the College of Medicine & Health Sciences of Arba Minch University, Ethiopia (Ref No IRB/84/2019; Date 12/12/2019) has approved the study. Participation in the study was entirely voluntary. Participants were given an all-rounded information sheet with the study’s objectives to read or were read loudly for those who could not read and asked to present themselves at a convenient specific day and time for the IDI if they agreed to participate. For the specific setting and study periods (1^st^ May to 31^st^ August 2020 for lesion cases & 1^st^ July to 31^st^ August 2021 for CL-scar cases), their oral consent was obtained before the interview. The participant’s identities were anonymized using the initials of their names and sub-village in the data to maximize the privacy of the participants’ identities. Feedback on the findings will be communicated to the study community once this article is published.

We have developed a detailed legal framework that guides the study’s implementation, ensuring it is ethical and relevant, and enhances both the quality and impact of the research (S1 Fig).

## Result

In this section, we describe the results of two data sets. We have used quotes to support the results. The respondent’s name initials, age, and sub-village are put between brackets for each quote.

### Sociodemographic characteristics of study participants

A total of ten people were included in the study of people with active CL lesions (Table 1). Seven were women and three were men. There were no reports of refusal or dropping cases from the study. Among the ten, six were parents of little children (five <15 years) who couldn’t explain themselves and five were patient interviewees. In CL Scar, there were nine participants, five being females and the rest being male (two are <17 years of age). For the age distribution, six were between the ages of 15 and 34, and four were 35 and older. A third of the participants had no formal education, while the rest had schooling ranging from third to tenth grade.

**Table 1:** Sociodemographic characteristics of the study participants with active CL lesion and CL scar, Ochollo, Ethiopia.

Variables	Active CL lesion (N = 10)			CL scar (N = 9)		
	Male	Female	Total (%)	Male	Female	Total (%)
**Age in years**						
15–24	2	3	5 (50)	2	-	2 (22.2)
25–34	-	1	1 (10)	2	1	3 (3.3)
35–44	-	1	1 (10)	-	1	1 (11.1)
45–54	-	1	1(10)	-	1	1 (11.1)
>55	1	1	2 (20)	-	2	2 (22.2)
**Religion**						
Protestant	2	4	6 (60)	3	3	6 (66.6)
Orthodox	1	3	4 (40)	1	2	3 (33.3)
**Occupation**						
Student	1	2	3 (30)	1	-	1 (11.1)
Farmer	1	-	1 (10)	1	-	1 (11.1)
House-wife	-	5	5 (50)	-	4	4 (44.4)
Weaving	1	-	1 (10)	2	-	2 (22.2)
Petty-trade	-	-	-		1	1 (11.1)
**Sub-village**						
Denkera (.DE.)	1	6	7 (70)	2	3	5 (55.5)
Kokima (KO)	-	-	-	2	2	4 (44.4)
Kancho (KA)	2	1	3 (30)	-	-	-
**Marital Status**						
Single	1	4	5 (50)	2	1	3 (33.3)
Married	2	3	5 (50)	1	4	5 (55.5)
Divorced	-	-		-		-
Widowed	-	-		-	1	1 (11.1)
**Educational status**						
Illiterate	-	2	2 (20)	-	3	3 (33.3)
1-6^th^ grade	2	1	3 (30)	1	2	3 (33.3)
7-8^th^ grade	0	1	1 (10)	3		3 (33.3)
9-12^th^ grade & above	1	3	4 (40)	-	-	-

Out of the ten participants in the CL lesion, eight had a formal education from as low as grade three up to grade tenth and two (female) were unable to read or write (never had formal education). On the contrary, there were only three participants who never had a formal education from the CL scar participants.

### Results of the data from people with an active lesion

**Fig 1:**
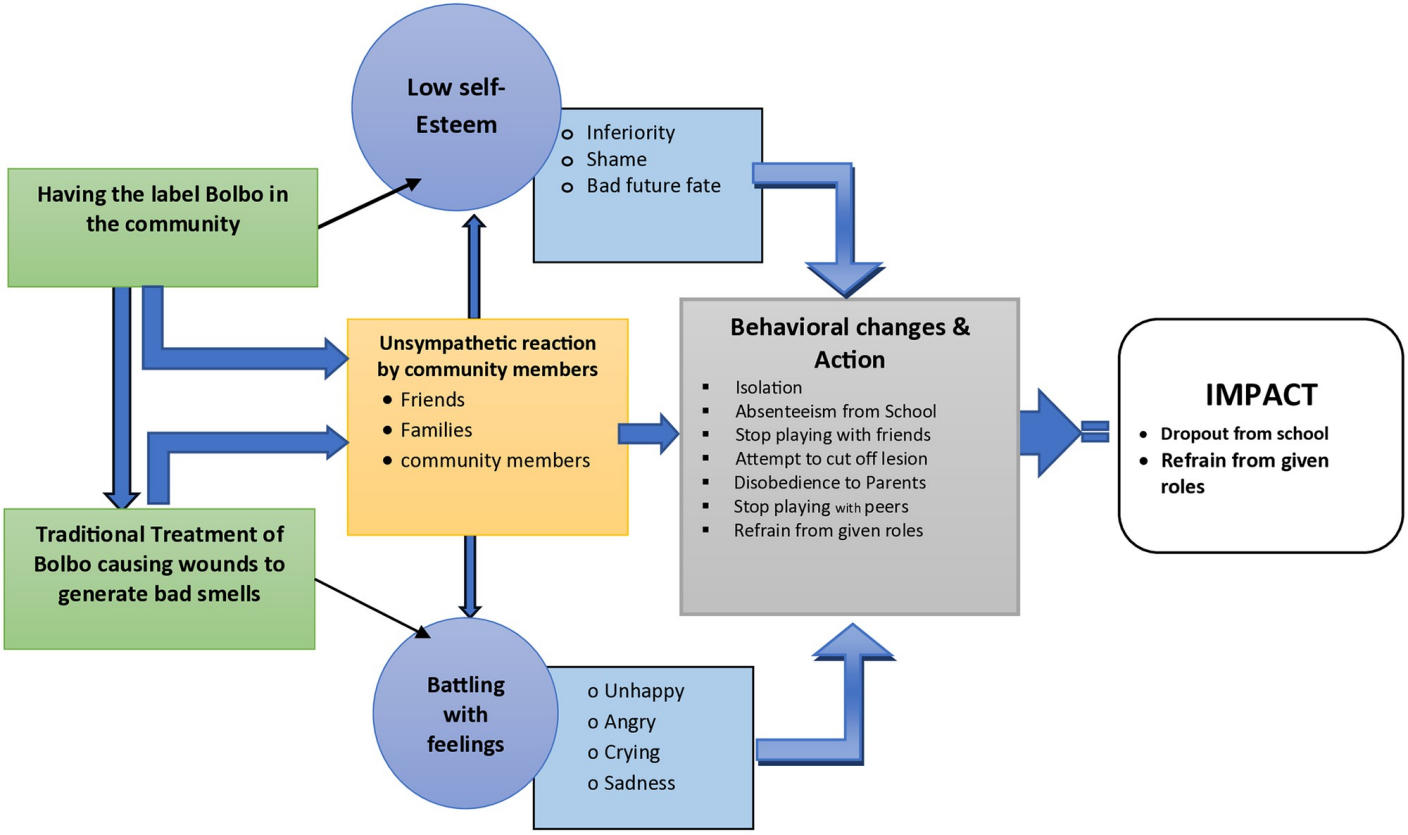
Data driven conceptual model of the processes of CL lesion-related stigma in Ethiopia.

Analyses of the lesion data have revealed seven interrelated themes contributing to the psychosocial problems the participants with CL lesions experienced: (1) **the presence of “Bolbo”** (2) **Traditional Treatment of Bolbo causing wounds to generate bad smells** (3) **unsympathetic external environment and reactions** (4) **low self-esteem the daily experiences,** (5) **Action** (6) **Behavioral changes**, and (7) **impact** of the lesion (See Fig 1 & S1 Table).

### People are labeled for CL lesions

The experiences of the participants showed the unfavourable views and misconceptions about CL and the impact it has on those who have visible lesions. CL is referred to as "Bolbo" or "Chaja" in several locations in the Gammo zone. Although they have no intrinsic meaning, these phrases have become associated with people who have CL lesions on their bodies. Individuals being labelled based on their CL lesions might lead to accepting a—misconception such as a “punishment from God” and bad attitudes. Participants described how they interpreted the source of CL and the sensation.

*“People do not understand that the person hasn’t acquired it willingly*. *A human being has to be honored and valued as a human being and should not be judged by the wound he or she has now”*

(FT, M, 39, SK).

One participant described a shoot of negative attitude that has outgrown the labelling of a person with CL as follows:

*“When someone says that you have a Bolbo, it is a shame for you*”

(EE, M, 54, KA).

### Traditional Treatment (TT) of CL generates bad smells

According to the participants, applying herbal medicine to the CL lesion has resulted in the formation of pus as well as a strong, unpleasant odour. This odour has caused worry, rage, and social stigma for those who are affected.

*“…… after the start of traditional treatment, the lesion holds pus*. *This pus is the cause of the beginning of a very bad odour*. *At this time, young children and youths begin to say “This person has a bad disgustingly smelling Bolbo”*. *This makes me worry and sometimes angry”*

(FB, M, 54, SK).

Another participant adds,

*“When the lesion is to heal after local treatment, it has pus and produces a very bad smell that one can smell as far as 20 meters*”

(EG, M, 45, TK).

### The unsympathetic external environment & reactions

The external environment has a profound impact on the experiences of people with active CL lesions. The surrounding environment is frequently unfriendly to them, and the bad stench caused by the application of traditional herbal medication to the lesions leads to negative sentiments from others. Participants mentioned difficulties in developing the unpleasant odor, which can cause embarrassment, discomfort, and worry. When interacting in society, this causes emotions of self-consciousness, humiliation, and anxiety.

*“Since I listened to these insulting words, I started really to feel sad and sometimes angry about the lesion on my face*”

(GG, M, 51, KA).

*“One more thing that every time bothers me is to see myself in a picture with a lesion on my face*. *I know how I look like and now like a person created again*, *I feel every time sad and say*, *it is the CL that made me be looked at differently*”

(EE, M, 54, KA).

The following are the day-to-day experiences of persons who had a foul-smelling infected CL lesion as well as others who merely had the "Bolbo".

*“So, I am afraid of the negative things the community has like rumouring, gossiping, talking like it is a new disease in the kebele, or as if I am the only first man in the kebele with Bolbo*”

(EE, M, 54, KA).

the everyday life experience in the society

*“My friends are insulting me and talking about me*. *They consider me as a lazy student and also rumoured about my parents that they wouldn’t take me for a traditional treatment”*

(FT, M, 39, SK).

CL has also been seen as a mark of a person’s locational identity,

*“When you walk along with your friends, people will see who is free of Bolbo and who has it*. *Then if they want to insult or identify you with the Bolbo you have, they say like “You with Bolbo”*. *This is a very bad way of expressing their dislikes towards people with Bolbo”*

(AD, 22, F, KA).

Participants with active CL have shared their life experiences of facing negative attitudes not only from the community but also from their family members, including parents and siblings. This indicates that the negative reflections and stigmatization extend beyond the external environment and permeate within the household.

*“Leave alone the outside people, even within the family member, her sisters and brothers didn’t like to see or have the bad-smelling when they sit together at home*. *They tell her, "Do not eat with us, do not sit among us, have a cover on your face”*

(EG, M, 45, TK).

### A cause for low self-esteem

The participants’ experiences shed light on the tremendous impact of CL lesions on individuals, leading to self-stigmatization, feelings of inadequacy, and unfavourable views from the community. Participants claim that those who contract "Bolbo" feel inferior, which contributes to social segregation. The existence of CL lesions causes people to think that they are inferior to others, which impacts their social interactions and relationships.

*“To my knowledge, a person who contracts “Bolbo” has the feeling of inferiority, that is why we have social segregation*”

(TS, M, 46, SK).

A person suffering from a CL lesion has an associated vision of a negative destiny and bad luck in life.

*“Children or people having Bolbo, especially after they know themselves, are a point of discussion and considered as someone who has got the bad fate of future”*

(EG, M, 45, TK).

Participants describe the community members’ unfavourable attitudes and expressions toward the individual with CL.

*“I know Bolbo has damaged my face*. *I said to myself and accepted that people were discriminating against me because of the Bolbo lesion I had on my face*. *Whenever I think about it, the way people reacted to me and said to me metaphorically always disturbs me*. *I feel guilty about Bolbo”*

(EE, M, 54, KA).

### The daily life experiences

The daily life experiences of individuals grappling with the challenges posed by their CL lesions. These narratives reflect genuine emotions and concerns, shedding light on the complex emotions and social interactions that influence their lives. Through their candid reflections, we gain a deeper understanding of how personal struggles intertwine with social dynamics, highlighting the impact of health conditions on their thoughts, feelings, and relationships. A participant said,

*“To be frank and to tell you the truth, I still have anger about why this cursed disease came to me after I was born and grew well*.*”*

(FB, M, 54, SK)

The negative point of view from friends

*“……my friends are insulting me and talking about me*. *They consider me as a lazy student and are also spreading rumours about my parents, wondering why they didn’t take me for local treatment…*.*”*

(FT, M, 39, SK).

### Action

All of the unfavourable attitudes and sentiments that patients have, along with the hostile external environment, can influence CL patients’ behaviour in general. Isolation was one of the behavioural changes reported by CL patients’ parents.

*“Now, people when their lesion has a bad smell, they know that people will behave badly towards you, then it is better to remain at home until the wound is healed”*

(EE, M, 54, KA).

*“He has isolated himself and his mood is also disordered*. *He also had anxiety due to the continued existence of lesions on his body*. *In addition to these*, *he had a feeling of shame and doubt about his future”*

(TM, M, 46, SK).

For school children, it has been a cause of absenteeism from school due to actions taken by parents.

*“We [a parent responded] have even kept her at home from going to school because she is very unhappy about going to school with the lesion”*

(EG, M, 45, TK).

The other action taken was refusing to play with friends.

*“……*..*self-isolation and she is reserved and shy because of the people’s behaviour towards her”*

(EG, M, 45, TK).

The aggressive actions were also a result of the negative attitudes of the people around them. A participant explains his extreme emotion and attempt.

*“Even after encountering an insult, I decided to cut off my lesion by knife myself”*

(GG, M, 51, KA).

*“We bought 10 modern hand sewing cloth needles to do that*. *We boiled them to make them clean*. *We started to pierce the swollen parts of the lesion*, *and when it bled*, *the blood was very dark”*

(EG, M, 45, TK).

### Behavioural changes and the overall impact

Patients with active CL face negative attitudes and an unsympathetic external environment, significantly impacting their well-being and behaviour. This has led to behavioural changes, including absenteeism from school for children.

*“My 8-year-old child has shown to be isolated from his school friends, crying and not even willing to go to school then after*. *He refused to even serve at home”*

(TM, M, 46, SK).

*“Children become passive both at home and at school*. *They tend to stop playing in some cases because of the friends talking about the disease”*

(EE, M, 54, KA).

*“We have even kept her home from schooling because she is very unhappy about going to school with the lesion”*

(EG, M, 45, TK).

**Fig 2:**
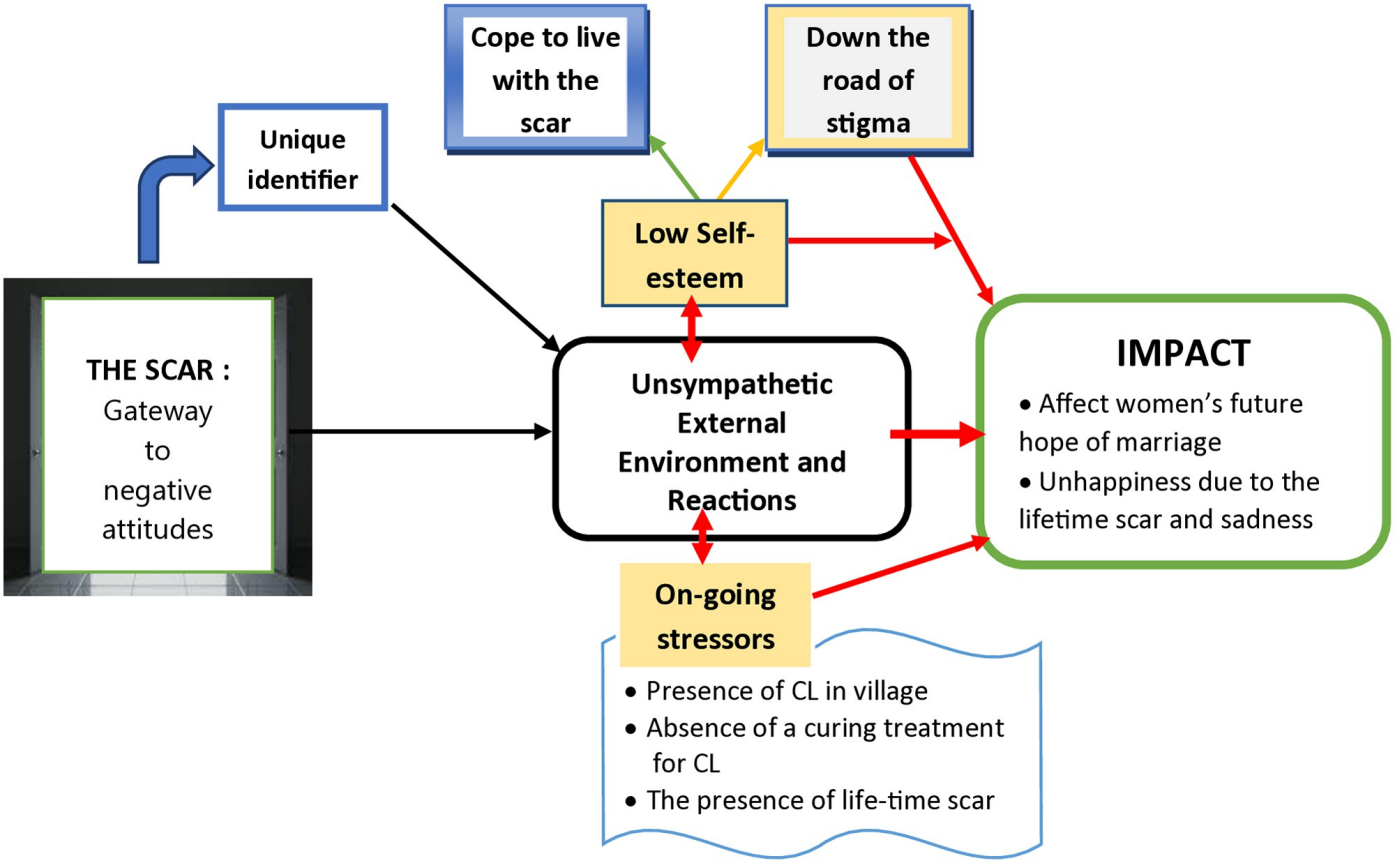
Data driven conceptual model of the processes of CL scar-related stigma in Ethiopia.

Analyses of the scar data have revealed seven interrelated major themes related to the psychosocial problems the participants with CL scars experienced: (1) the **“Scar” as the gateway for negative attitudes** (2) **Unsympathetic external environment and reactions** (3) **Low self-esteem** (4) **On-going stressor,** (5) **Cope to live with the scar,** (6) **Down the road of stigma**, and (7) **impact** of the scar (See Fig 2 & S2 Table).

### The scar: Gateway to negative attitudes

The presence of the scar is identified as the fundamental trigger for the development of negative psychosocial attitudes in individuals with CL scars (Fig 2). Participants explained that the scar serves as a unique identifier, representing their experience and belonging. The scar becomes an integral part of their identity, even shaping their perception of themselves in the future. Participants expressed concerns about the scars and the challenges they face in their day-to-day experiences. They feel bad about the scar and question why they have this disease and why they look different from their friends.

*“Because our children will ask us when they grow older and we have to do our best to avoid the future guilty feeling due to not trying our best now*. *The scar will be like an identity about from where we belong even in the future”*

(MG, F, 45, DE).

*“But*, *if it is what I feel about the scar now*, *it scares me*, *I feel bad*. *I feel bad about the scar*. *I feel like*, *why did I get this disease*? *Why do I look different from my friends*? *Why should others insult me for the scar*? *All these thoughts come into my mind causing discomfort and disappointment”*

(EA, M, 16, DE).

A17-year-old female participant expresses

*“Whereas for the scar, you will start feeling about it at some point and then may continue throughout your lifetime or depending upon your encounters and how you feel about them”*

(HK, F, 17, DE).

### Unsympathetic external environment and reactions

People’s social environments and interactions have a vital role in determining the reactions of the people with scars will have towards other people, the environment itself and all about life. The unaccommodating and unsympathetic external environment can be influential to change the participants’ attitude to accept that can affect the way they see themselves. It is expressed by participants as follows.

*“But every time I feel bad*. *I don’t like the scar*. *I feel about the scar of course*. *How? When I look at my picture, I see myself with a scar on my face”*

(HK, F, 17, DE).

People who are perpetually exposed to such negative stimuli can go either way: coping with the unreceptiveness and accepting the reality to live with it or going down the road of stigma.

Participants expressed scar as an ongoing stressor.

*“You cannot remove the scar from the face*. *It is not removable*. *When I see myself in the mirror or see my picture, the scar is visible*. *When I realize that, I feel bad*. *It gives me stress*.*”*

(ET, F, 17, DE)

Participants who accepted the reality and lived with the scar had to say about coping with the unreceptiveness environment.

*“I only feel the scar when I look in a mirror sometimes*. *It’s fine*. *I can’t change it and this has already happened*. *I don’t feel ashamed, guilty, or anything”*

(AL, F, 25, KA).

*“*..*The way I look towards myself is ok*. *I am healthy*, *I can do anything*, *I am married*, *I live peacefully with my community*.*… I never had any worries about getting good company (wife)*, *and thank God I had*. *I am happy”*

(MM, M, 60, DE).

### Low self-esteem

In the context of low self-esteem, the participant’s emotional response is evident. They express a sense of sadness and a feeling of being disconnected from their ideal self, likening it to a less desirable version of themselves. A participant says the following,

*“I feel sad about it; This is like I am with the second version of myself*.*”*

(AL, F, 25, KA)

*“I experienced a deep sense of distress*, *concern*, *and embarrassment over the situation*. *I was born in good health*, *but unfortunately*, *my face has been altered due to Bolbo scar I developed”*

(AA, F, 45, DK)

### The scar is an ongoing stressor

The participants describe the distress caused by the constant reminder of the scar’s presence, leading to feelings of shame and worry. The inescapability of the scar’s visibility, whether in mirrors or photographs, amplifies their negative emotions, resulting in a significant source of stress. (MM, M, 60, DE) provide insights into the emotional impact of these visible reminders.

*“Because the scar is on my nose and I see it every time when I open my eyes, it is in front of me! This worries me now*. *I can see it*. *It is something that I always feel ashamed of when people talk about what has happened to my nose*.*”*

(MM, M, 60, DE).

### Coping to live with the scar

For AL, a female participant aged 25 and coming from the KA sub-village, coping with the scar involves an accepting mindset. When she sees her reflection, the scar only has a small amount of meaning. She recognizes that it will always be there and that it was an uncontrollable previous circumstance. She doesn’t approach it with feelings of guilt or shame but with self-compassion and apathy.

*“I only feel about the scar when I look at a mirror sometime*. *It’s fine*. *I can’t change it and this has already happened*. *I don’t feel ashamed, guilty or anything*.*”*

(AL, F,25, KA)

### Impact of CL scar

As shown in Fig 2, individuals living with the CL-scar and have low self-esteem and are unable to cope with living with the scar might walk down the route of shame, as some participants did. The scar had a substantial influence on their life, influencing their performance, desire for marriage, and general self-satisfaction. The scar also had a skewed effect on females, who were subjected to more societal pressure and shame over their future marital possibilities. These findings emphasize the need to receive assistance and understanding while dealing with the psychological and social repercussions of scars in a hostile setting. The presence of a scar on the nose becomes an inescapable source of stress and unhappiness, affecting daily experiences and self-esteem. Similarly, (MM, M, 60, DE) speaks to the scar’s enduring influence, perceiving it as an inextricable part of his life, constantly in his sight.

*“The stress, if I have to say it is the scar on my nose*. *I can’t avoid it from my eyes and I say, it is always with me*. *It goes wherever I go and it appears before anything whenever I open my eyes*. *I think this is something that will stay with me*.*”*

(MM, M,60, DE).

The impact extends beyond emotional well-being; (FP, M, 22, KA) outlines how scars have led to significant life challenges. People’s job performance, marital prospects, and educational opportunities have been hindered by the scars, reflecting the broader repercussions on personal and social aspects of life.

*“…my job performance……*.*my interest of getting married is also affected, which I cannot dare to ask a girl to marry me with this disease*. *I never attended any school and have no idea of starting schooling after such age*.*”*

(FP, M,22, KA).

The effects of the scar had also an impact in terms of gender as an important marker that would determine their future marriage affecting more females than boys. A female participant expresses the following:

*“For men, leave alone a scar, why not he is having a disability, no problem of getting a wife*. *But, for the girls, it is so bad and worrying for them in this situation [scar]*. *If she doesn’t get a husband, then the unfortunate woman who didn’t find a partner will have all the blame and insults from the community like, a “stand-alone woman”, and a leftover woman*. *So, more worries in girls than in boys”*

(AL, F, 25, KA).

## Discussion

This study investigated the psychosocial implications of CL, a significant public health concern with physical, social, and psychological ramifications in one of the endemic foci of southern Ethiopia, employing a descriptive phenomenological design. The research aimed to comprehend the experiences of individuals with CL lesions and scars with an important highlight on how CL-related stigma is constructed and the resultant impacts to help for designing appropriate prevention strategies [29–31].

In this study, both participants with CL lesions and scars had the belief that most people in the community and individuals looking at them had held stigmatizing attitudes or beliefs towards them. This confirms the action-oriented type of stigma as mentioned [14]. It is the perception that society as a whole embraces negative views or stereotypes towards people with CL lesions or scars. In this study, this perception had a significant impact on individuals’ self-esteem and well-being.

The CL-related (health-related) stigma [32] in Ochollo had significant psychological and social consequences for individuals. It has led to negative self-perceptions, low self-esteem, and internalized stigma, where stigmatized individuals consider themselves less worthy than others [31]. In this research, stigmas manifest themselves in various ways, such as anticipation of their stigmatized identity, and internalized negative stereotypes associated with the individual’s stigmatizing identity (lesion or scar) resulting in feelings of shame, guilt, and fear as described. These are consistent with the descriptions given by researchers as Perceived Public stigma, Self-stigma and affiliative stigma [33]. The impact of stigma on an individual’s life is described in terms of fear of losing a partner, especially in women and rejection by families [34].

It also affected the stigmatized group (those with CL lesions and scars) by forcing them to take action such as self-social and school exclusion and loss of future life opportunities [29,30].

In most circumstances, patients with CL lesions in this study had visited a traditional healer for one or repeated application of a medicinal plant, which is in line with a study on the use of traditional medicines in Ethiopia [35,36].

The stigma associated with disfiguring NTDs such as CL is well-documented, and it has been shown to have a significant influence on hindering disease control efforts by affecting treatment adherence and the effectiveness of public health interventions [37]. The understanding of stigma in NTDs can serve as a tool for expanding awareness of the illnesses and their societal effects, monitoring the degree of stigma over time, and analyzing stigma changes considering cultural validity, including increasing understanding of the disease and their social impact [38].

Those who visited the traditional healer though expressed the curing ability and their trust, they also mentioned that following the application of a medicinal plant, a foul-smelling (infection) is an inevitable phenomenon to go through which we found as a major starter for the whole negative attitude towards people with the lesion. An aspect of inequitable psychological impact that affects gender differentially has also been an important aspect revealed in this study. The reason why a current lesion is an ongoing stressor to female participants and their parents was due to the fear of the unavoidable permanent scar that would damage their faces. This has impacted their psychology as a determining condition for their future marriage life, a woe besides a change in aesthetics only.

In our study, a low self-esteem outlook manifested by anxiety, disappointment, fear and sadness has been documented as it is in studies of other NTDs. The affected gender in this study were females as the lesion and the resultant scar are serious concerns and worries about their future chance of finding a partner for marriage due to the disfiguring scar. This has also been similarly confirmed in other studies [39]. Family members had contributed in stigmatizing their child. In our study, while few participants from the lesion group have tried to accept positively the situation and wait and see the outcome, those from the scar had the contemplation of getting the scar removed by a possible surgical intervention in way out of low self-esteem due to CLS, as Goffman described [12].

## Conclusion and recommendations for action

By addressing these policy implications, recommendations that can be put into practice and research gaps, stakeholders can work towards reducing the stigma associated with CL and improving the well-being of affected individuals and communities in Ethiopia and beyond.

Based on our study, we recommend the following for the implementation of good practices to address and mitigate the effects of stigmatization associated with CL:

When evaluating policy implications, it is imperative to emphasize the adoption of an integrated approach, as recommended by the World Health Organization [40]. This comprehensive strategy is essential as it encompasses the physical, mental, and social dimensions of Neglected Tropical Diseases, particularly Cutaneous Leishmaniasis, thereby effectively addressing pain, emotional distress, and social stigma concurrently.

Delving into gender perspectives, it is critical to acknowledge the disproportionate impact of Cutaneous Leishmaniasis on women and girls. Interventions need to specifically target gender-related exposure, socioeconomic discrepancies, and other gender dynamics. Adapting strategies from a gender perspective is crucial in effectively combating the stigma associated with Cutaneous Leishmaniasis [39].

Utilizing Indigenous Community-Based Networks is essential for engaging with communities and aligning with existing social practices. Leveraging existing Indigenous community-based social networks and support organizations, such as coffee-drinking cultures and women’s support groups, is crucial in reducing the stigma around CL. These networks provide crucial opportunities for empowerment, advocacy, and the sharing of experiences, creating a supportive environment that significantly contributes to the overall well-being of the community [41].

To address misconceptions, alleviate anxiety, and reduce the stigma surrounding CL and other NTDs, it is crucial to implement more comprehensive health promotion and education programs. By improving awareness and understanding, these initiatives can play a significant role in enhancing the reporting of CL cases [42].

Incorporating active surveillance of CL into routine Primary Health Care packages is crucial for effectively monitoring and identifying cases of this disease. By implementing active surveillance methods, healthcare providers can promptly detect and diagnose cases of CL within the community, allowing for early intervention and treatment. This proactive approach not only helps minimize the physical suffering experienced by those affected but also reduces the economic burden and psychological impact on individuals and communities. This integrated approach to active surveillance and early case detection plays a key role in controlling the spread and impact of cutaneous leishmaniasis.

### Future research suggestions

Further research is crucial to conducting an ethnographic exploration of traditional treatment for CL. Understanding the lived experiences, efficacy, and cultural context of traditional healing methods can provide valuable insights for the development of more comprehensive healthcare strategies for managing CL.

The current study is deficient in providing evidence regarding the economic impacts of CL. There is a need for future research to thoroughly investigate the economic burden experienced by individuals and communities affected by CL. This comprehensive investigation would be instrumental in informing resource allocation and shaping policy decisions related to CL

### Limitations of the study

As the principal investigator, I have observed that the medicinal plant used by local traditional healers to treat CL has the potential to heal CL. Therefore, we believe that further research should be conducted to explore the potential of the herb for new drug discovery and from an ethnographic perspective. This study also lacks evidence about the economic impacts of CL. However, in its state, this paper could give a very good insight into CL lesions and related scar-related stigma in Ethiopia to design an appropriate strategy to mitigate the prevention of CL.

## Supporting information

S1 Table Themes and sub-themes identified from participants with CL lesion, southern Ethiopia, 2021. (DOCX)

S2 Table Themes and sub-themes identified from participants with CL scar, southern Ethiopia, 2021. (DOCX)

S3 Table Consolidated criteria for reporting qualitative research (COREQ): A 32-item checklist for interviews and focus groups used to report our findings. (DOCX)

S1 Fig Compliance of research with legal frameworks. (TIF)

S2 Fig A code-tree for CL lesion: Inductive. (PDF)

S3 Fig A code tree for CL scar- themes and sub-themes. (PDF)
