# Supplementary material for: Unveiling the hidden burden: Exploring the psychosocial impact of cutaneous leishmaniasis lesions and scars in southern Ethiopia
Source: PLoS One. 2025 Feb 5;20(2):e0317576. doi: 10.1371/journal.pone.0317576 (PMC11798448; doi:10.1371/journal.pone.0317576)
Supplement: S1 Table — (DOCX) [file pone.0317576.s001.docx]

**Supporting Information**

**S1 Table. Themes and sub-themes identified for participants with CL lesion, southern Ethiopia,** **2021.**

| **Sno** | **Main themes** | **Sub-themes** |
| --- | --- | --- |
| **1** | **Having the label Bolbo in the community** | The lesion is a reason to lessen human value |
|  |  | Shame |
| 2 | **Traditional Treatment of Bolbo causing wounds to generate bad smells** | The bad odour that comes after some time of getting the traditional treatment |
| **3** | **Unsympathetic reactions** | Friends |
|  |  | Families |
|  |  | Community members |
|  | **Low self-esteem** | Inferiority |
|  |  | The bad fate of the future |
|  |  | Shame |
| **4** | **Battling with the feelings** | Unhappy |
|  |  | Angry |
|  |  | Crying |
|  |  | Sadness |
| **5** | **Action** | Isolation |
|  |  | Absenteeism from school |
|  |  | Stop playing with friends |
|  |  | Attempt to cut off the lesion |
| **6** | **Behavioural changes** | Disobedience |
|  |  | Stop Playing with others |
| **7** | **Impact** | Dropout from school |
|  |  | Refrain from given roles |
