# Supplementary material for: Unveiling the hidden burden: Exploring the psychosocial impact of cutaneous leishmaniasis lesions and scars in southern Ethiopia
Source: PLoS One. 2025 Feb 5;20(2):e0317576. doi: 10.1371/journal.pone.0317576 (PMC11798448; doi:10.1371/journal.pone.0317576)
Supplement: S2 Table — (DOCX) [file pone.0317576.s002.docx]

**S2 Table. Themes and sub-themes identified for participants with CL scar, southern Ethiopia, 2021.**

| **S no.** | **Main themes** | **Sub-themes** |
| --- | --- | --- |
| **1** | **The SCAR: Gateway to negative attitudes** | The scar is a unique identifier |
| **2** | **Unsympathetic external environment and reactions** | Other’s Perceptions and attitudes towards people with CL scar |
| **3** | **Low self-esteem** | The way people see themselves |
| **4** | **On-going stressor** | I worry about my future |
| **5** | **Cope to live with the scar** | Adaptability and endure to be familiar with the other’s unreceptiveness |
| 6 | **Down the road of stigma** | Unable to cope with the scar |
| **7** | **Impact** | Lifetime unhappiness due to CL's scar |
|  |  | CL Scar affects women more |
