# Supplementary material for: Unveiling the hidden burden: Exploring the psychosocial impact of cutaneous leishmaniasis lesions and scars in southern Ethiopia
Source: PLoS One. 2025 Feb 5;20(2):e0317576. doi: 10.1371/journal.pone.0317576 (PMC11798448; doi:10.1371/journal.pone.0317576)
Supplement: S3 Table — (DOCX) [file pone.0317576.s003.docx]

**S3 Table: Consolidated criteria for reporting qualitative research (COREQ): A 32-item checklist for interviews and focus groups used to report our findings.**

| **Domain 1: Research team and reflexivity Personal Characteristics** | | | **Responses** |
| --- | --- | --- | --- |
| **1. Interviewer/facilitator** | Which author/s conducted the interview or focus group? | The first round of ID interviews for lesion cases was done by AS (a master's student) and all other ID interviews were done by BM (the author) | |
| **2. Credentials:** | What were the researcher’s credentials? e.g. PhD, MD | Two data collectors: BM (PI) is a PhD student with a master's in Global Health; AS was a student and now graduated with a Master's in Public Health;. | |
| **3. Occupation:** | What was their occupation at the time of the study? | BM was PhD student on leave; AS was an MPH student at Arba Minch University, | |
| **4. Gender:** | Was the researcher male or female? | BM & AS are Male. | |
| **5. Experience and training Relationship with participants** | What experience or training did the researcher have? | PI has undertaken training in Epidemiology-Biostatistics and Qualitative research methods (EBQ) course at the University of Antwerp, Belgium | |
| **Relationship with Participants** | | | |
| **6. Relationship established** | Was a relationship established prior to study commencement? | No prior relationship was established between the researchers and participants, except for informing about the research objectives and consent. | |
| **7. Participant knowledge of the interviewer** | What did the participants know about the researcher? e.g. personal goals, reasons for doing the interviewer research | Participants knew from where the researchers had come, who he is and the purpose of the research. | |
| **8. Interviewer characteristics** | What characteristics were reported about the interviewer/facilitator? e.g. Bias, assumptions, reasons and interests in the research topic. | The interviewers and research co-authors had an interest in the research. | |
| **Domain 2: study design** | | | |
| **Theoretical framework** | | | |
| **9. Methodological orientation and Theory** | What methodological orientation was stated to underpin the study? e.g. grounded theory, Theory Participant selection discourse analysis, ethnography, phenomenology, content analysis | The study design followed a descriptive phenomenology study and was guided by thematic analysis (TA). | |
| **10. Sampling** | How were participants selected? e.g. purposive, convenience, consecutive,  snowball | A purposive sample for both patients with active CL lesions and CL scars (ten for lesions and nine for scars) was recruited from the village. The researchers contacted the traditional healers to find lesion cases and scar cases were identified from a case registry of a previous study by the PI. | |
| **11. Method of approach** | How were participants approached? e.g. face-to-face, telephone, mail, email | Candidate participants were approached by village healers. After receiving an information sheet and giving consent, an interview was scheduled at their convenience. | |
| **12. Sample size** | How many participants were in the study? | Ten for lesions/Nine for scars have participated in the study. | |
| **13. Non-participation Setting** | How many people refused to participate or dropped out? Reasons? | We never had a record of refusals. | |
| Research Setting | | | |
| **14. Setting of data collection** | Where was the data collected? e.g. home, clinic, workplace | All the In-depth Interviews for both Lesion and scar cases were conducted at each participant's residence. | |
| **15. Presence of non-participants** | Was anyone else present besides the participants and researchers? | A researcher and a trusted Interpreter who translated metaphors and proverbs to the PI was present. | |
| **16. Description of sample Data collection** | What are the important characteristics of the sample? e.g. demographic data, date | Demographic information was collected about the participants | |
| **17. Interview guide** | Were questions, prompts, and guides provided by the authors? Was it pilot-tested? | Interview materials with queries were created and used throughout the session. These were thoroughly reviewed by the study team. | |
| **18. Repeat interviews** | Were repeat interviews carried out? If yes, how many? | No repeat interviews are required. | |
| **19. Audio/visual recording** | Did the research use audio or visual recording to collect the data? | Interviews were audio recorded. | |
| **20. Field notes** | Were field notes made during and/or after the interview or focus group? | Yes. | |
| **21. Duration** | What was the duration of the interviews or focus groups? | The average time is 35 minutes. | |
| **22. Data saturation** | Was data saturation discussed? | Yes, interviews were stopped after we realized that no more new ideas had come. | |
| **23. Transcripts returned** | Were transcripts returned to participants for comment and/or correction? | Transcripts were not returned to participants as most were illiterate. | |
| **Domain 3: Analysis and Findings** | | | |
| **Data analysis** | | | |
| **24. Number of data coders** | How many data coders coded the data? | Two researchers coded independently | |
| **25. Description of the coding tree** | Did the authors describe the coding tree? | The code tree is attached as appendices. | |
| **26. Derivation of themes** | Were themes identified in advance or derived from the data? | Themes were derived from the data. | |
| **27. Software** | What software, if applicable, was used to manage the data? | NVivo 12 | |
| **28. Participant checking Reporting** | Did participants provide feedback on the findings? | Community members were communicated about the result at a workshop | |
| **29. Quotations presented** | Were participant quotations presented to illustrate the themes/findings? Was each quotation identified? e.g. participant number | Yes, we used the quotes from the participants to illustrate the findings and create the theoretical frameworks. | |
| **30. Data and findings consistent** | Was there consistency between the data presented and the findings? | Yes, we believe that we have reported the study findings in a clear, consistent manner to accurately reflect the data that have been collected | |
| **31. Clarity of major themes** | Were major themes clearly presented in the findings? | Yes, the major themes are clearly presented in the result section, | |
| **32. Clarity of minor themes** | Is there a description of diverse cases or a discussion of minor themes? | The minor, but important descriptions were managed to be incorporated into the CL-related stigma theoretical frameworks of both the lesion and scars. | |

**Source of tool:**

Tong A, Sainsbury P, Craig J. Consolidated criteria for reporting qualitative research (COREQ): a 32-item checklist for interviews and focus groups. Int J Qual Health Care. 2007;19(6):349-57. doi: 10.1093/intqhc/mzm042. PMID: 17872937.
