# Supplementary figures and images for: Unveiling the hidden burden: Exploring the psychosocial impact of cutaneous leishmaniasis lesions and scars in southern Ethiopia
Source: PLoS One. 2025 Feb 5;20(2):e0317576. doi: 10.1371/journal.pone.0317576 (PMC11798448; doi:10.1371/journal.pone.0317576)

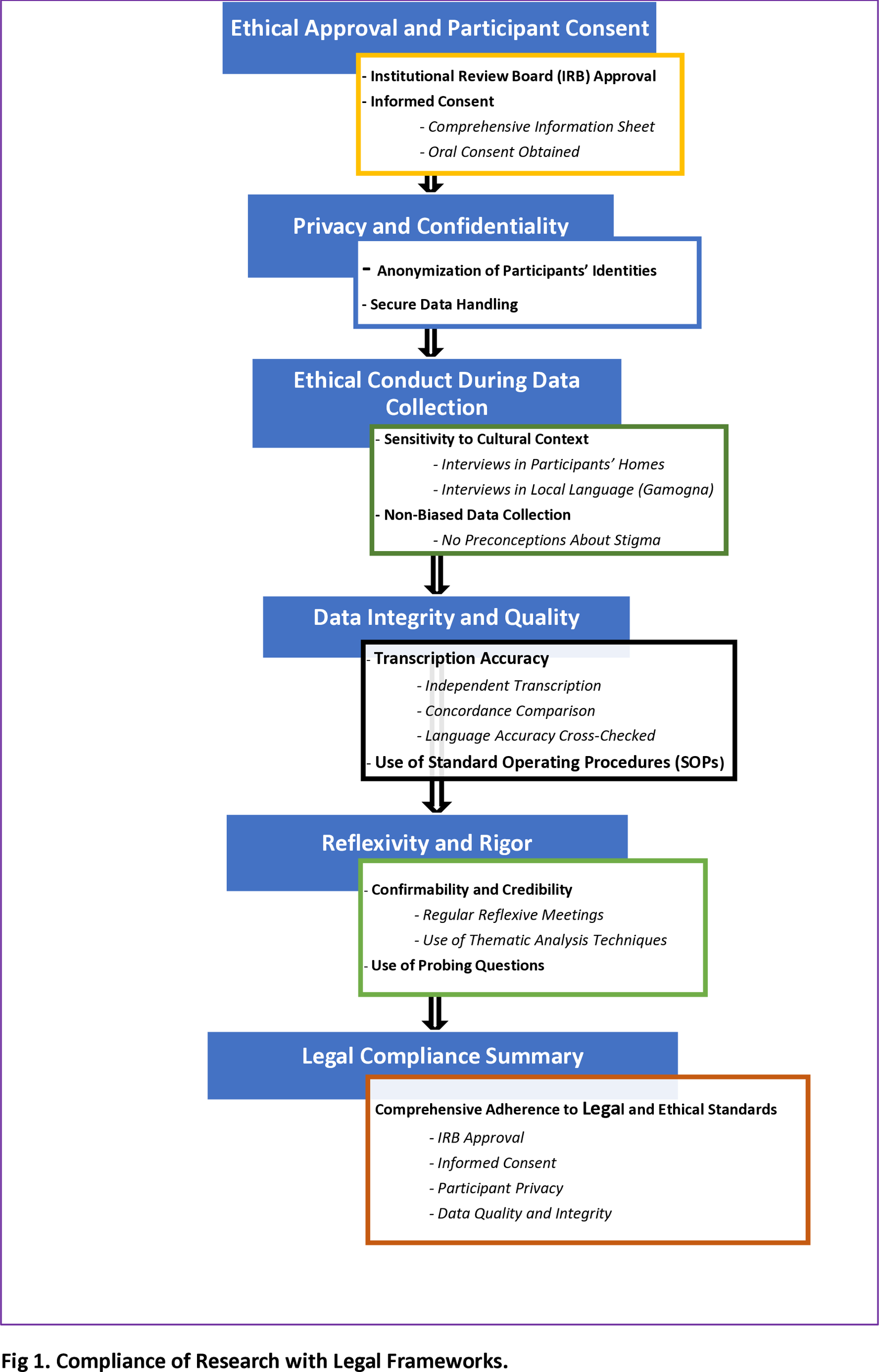

Supplement: S1 Fig — (TIF) [file pone.0317576.s004.tif]

**S2 Fig: A- Code-tree for CL lesion: Inductive**

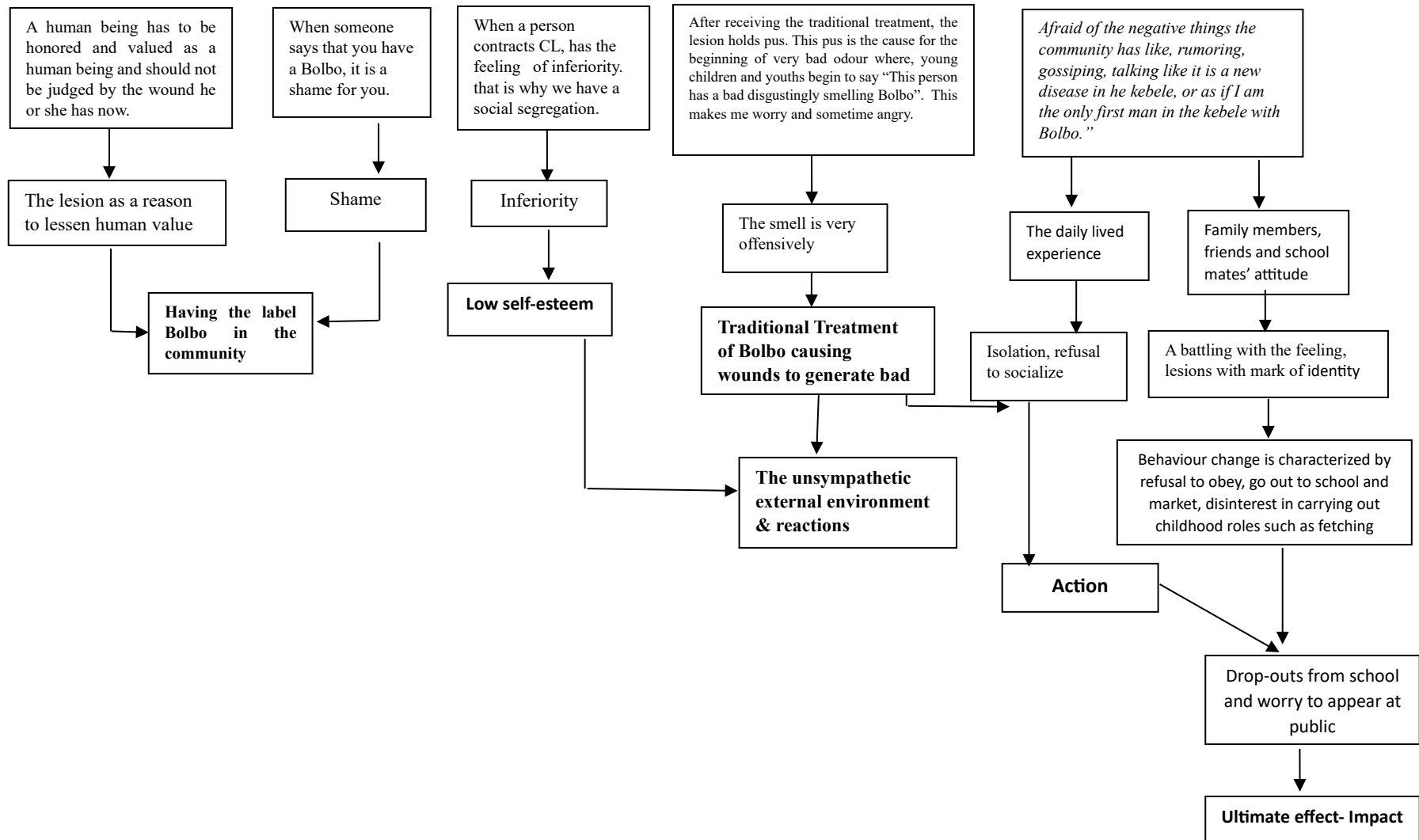

Supplement: S2 Fig — (PDF) [file pone.0317576.s005.pdf]

## The code Tree for CL Scar- themes and Sub-themes

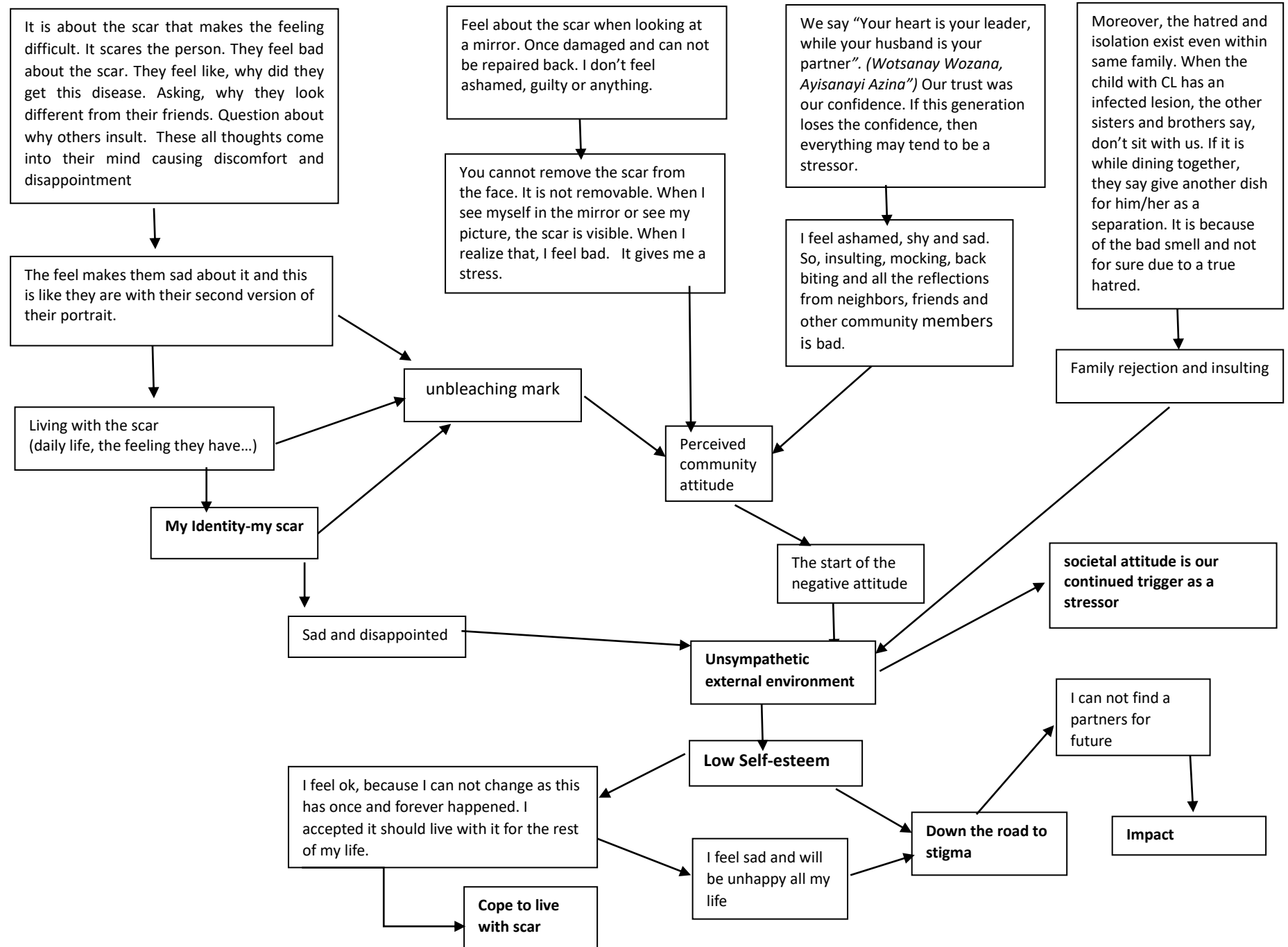

Supplement: S3 Fig — (PDF) [file pone.0317576.s006.pdf]
